# Integrating Retrogenesis Theory to Alzheimer's Disease Pathology: Insight from DTI-TBSS Investigation of the White Matter Microstructural Integrity

**DOI:** 10.1155/2015/291658

**Published:** 2015-01-20

**Authors:** Gilberto Sousa Alves, Viola Oertel Knöchel, Christian Knöchel, André Férrer Carvalho, Johannes Pantel, Eliasz Engelhardt, Jerson Laks

**Affiliations:** ^1^Translational Psychiatry Research Group, Department of Clinical Medicine, Federal University of Ceara, Rua Professor Costa Mendes 1608, 4° Andar, Rodolfo Teófilo, 60430140 Fortaleza, CE, Brazil; ^2^Institute for General Medicine, Goethe University, 60590 Frankfurt am Main, Germany; ^3^Department of Psychiatry, Psychotherapy and Psychosomatics, Goethe University, 60528 Frankfurt am Main, Germany; ^4^Alzheimer's Disease Center, Federal University of Rio de Janeiro (UFRJ), 22290140 Rio de Janeiro, RJ, Brazil; ^5^Department of Cognitive and Behavior Neurology, Federal University of Rio de Janeiro (UFRJ), 22290140 Rio de Janeiro, RJ, Brazil; ^6^Centre for Study and Research on Aging, Instituto Vital Brazil, 22451000 Rio de Janeiro, RJ, Brazil

## Abstract

Microstructural abnormalities in white matter (WM) are often reported in Alzheimer's disease (AD) and may reflect primary or secondary circuitry degeneration (i.e., due to cortical atrophy). The interpretation of diffusion tensor imaging (DTI) eigenvectors, known as multiple indices, may provide new insights into the main pathological models supporting primary or secondary patterns of WM disruption in AD, the retrogenesis, and Wallerian degeneration models, respectively. The aim of this review is to analyze the current literature on the contribution of DTI multiple indices to the understanding of AD neuropathology, taking the retrogenesis model as a reference for discussion. A systematic review using MEDLINE, EMBASE, and PUBMED was performed. Evidence suggests that AD evolves through distinct patterns of WM disruption, in which retrogenesis or, alternatively, the Wallerian degeneration may prevail. Distinct patterns of WM atrophy may be influenced by complex interactions which comprise disease status and progression, fiber localization, concurrent risk factors (i.e., vascular disease, gender), and cognitive reserve. The use of DTI multiple indices in addition to other standard multimodal methods in dementia research may help to determine the contribution of retrogenesis hypothesis to the understanding of neuropathological hallmarks that lead to AD.

## 1. Introduction

Alzheimer's disease (AD) is one of the most prevalent neurodegenerative disorders in the elderly which is estimated to affect tens of millions of people worldwide [1]. A recent study estimates that dementia shall affect over 81 million individuals worldwide by 2040 [2]. The progression of clinical-pathological correlations of AD can be understood in terms of disconnection syndromes and functional distributed networks underlying cognitive abilities [1, 3].

The investigation of AD neuropathology and its relation to cognitive decline, previously restricted to postmortem studies, has developed substantially with the advent of neuroimaging techniques in the last decades. Early neuroimaging studies on AD were focused on volumetric based morphometric techniques and region of interest investigations (ROI), which were performed through image registration and smoothing [4]. Progressively, conventional ROI approaches have been replaced by whole brain diffusor tensor imaging (DTI) analysis, which offers higher accuracy for white matter (WM) registration between subjects. DTI enables the definition of major WM tracts and their trajectories and also WM microstructure [1], thus providing a comprehensive investigation of brain circuitry integrity. DTI is sensitized to the random motion of water molecules as they interact within tissues, thus reflecting characteristics of their immediate structural surroundings. One of the most frequently employed DTI methods is the tract-based spatial statistics (TBSS) [5], which represents an effort to overcome some limitations of conventional ROI method [6, 7], including tract misalignment and variance effects in brain atrophy and partial volume estimations [8].

The evidence base gathered by DTI investigations in the last decade has helped to better define the pathological cascade underlying AD [6]. Diffusion studies on AD were primarily focused on the pattern of lesions distribution, the localization of DTI changes, the distribution of disrupted networks, and the nature of microstructural pathology. Regardless of DTI sensitivity in assessing WM microstructural changes, differences in diffusion patterns across clinical groups may be challenging to interpret [1]. Several studies reported DTI changes in the parahippocampus, hippocampus, posterior cingulum, and splenium even at the MCI stage [9–13]. Widespread areas of DTI abnormalities may also be observed in AD. It has been estimated that the whole brain may present a mass reduction of nearly 3-4% per year [14].

**Figure 1:**
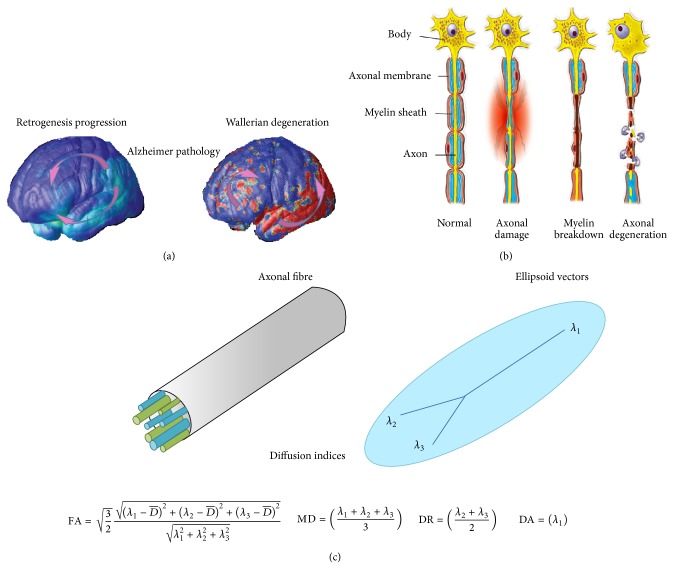
(a) Wallerian degeneration occurs as a secondary product of gray matter loss, while retrogenesis hypothesis outlines the degeneration of late-myelination fibers in neocortical areas. The Wallerian degeneration model postulates a posterior-anterior gradient of fibre degeneration (right side, arrows); the normal myelinisation occurs throughout the first life decades, beginning at dorsal brain and reaching neocortical areas at end stages (right side, arrows). According to the retrogenesis model, neocortical fibers are those more likely to suffer early degeneration by AD; (b) myelin breakdown and axonal damage are one of the key pathological mechanisms underlying white matter microscopic lesions (b). (c) A projection of the ellipse onto the three main axes (*λ*_1_, *λ*_2_, *λ*_3_) or eigenvectors. The main DTI indices of fractional anisotropy (FA) and axial (DA), radial (DR) and mean (MD) diffusivity are based on the eigenvector calculations (bottom).

On the microstructural level, WM abnormalities in AD may be interpreted as myelin breakdown and axonal damage [15]. Different pathological models have been suggested to account for these microstructural alterations: retrogenesis and Wallerian degeneration. Retrogenesis assumes primary white matter atrophy through myelin breakdown and axonal damage [15–18]. It has been suggested that fibers more susceptible to neurodegeneration due to the retrogenesis process are those with small-diameter corticocortical axons [19–21], namely, from the temporal lobe and neocortical areas. Conversely, the Wallerian degeneration assumes secondary white matter atrophy due to cortex degeneration [22]. Evidence favouring the Wallerian degeneration or the retrogenesis remains disputed [23]. For instance, neuronal disruption at predementia stages may not solely account for Wallerian degeneration and there are anatomical regions where the retrogenesis hypothesis might better explain WM atrophy (Figure 1). Moreover, the corpus callosum may be susceptible to AD and, depending on its anatomical localization, DTI changes would be associated either with retrogenesis or Wallerian degeneration [8]. Previous studies reported a correlation between gray matter (GM) temporal atrophy and the reduced volume of CC posterior segments [15], while in others cortical atrophy failed to show an association with anterior CC fibers [15]. In fact, it has been also demonstrated that the genu of the CC is a region where fibers myelinate later in neurodevelopment [24]; this region contains the highest density of small diameter fibers, whereas fibers of the splenium of the CC myelinate earlier on life [24].

The overarching aim of this comprehensive review is to summarize the main etiological mechanisms associated with AD neuropathology, based on the most recent alternative explanation, the retrogenesis hypothesis. The contribution of DTI studies to the understanding of the retrogenesis model is critically analyzed, as well as the clinical significance of the main DTI proxies (described in detail below) for the interpretation of neuropathological mechanisms involved in neuronal disruption, namely, axonal damage and myelin breakdown [25] (Figure 1). Additionally, patterns of neurodegeneration will be discussed in relation to risk factors, progression of lesions along disease course, DTI changes, and the influence of retrogenesis model on regional tracts. Finally, the interaction between late-myelinating alterations, amyloid deposition, and vascular factors in AD is reviewed.

## 2. Methods

A review of the literature was performed from 2004 to 2014 through searches in the electronic databases PubMed (http://www.ncbi.nlm.nih.gov/pubmed/), Institute for Scientific Information Web of Knowledge (http://www.isiknowledge.com), and EMBASE (http://www.embase.com), using the following terms: “retrogenesis,” “diffusion tensor imaging,” “DTI,” “Alzheimer's disease,” “mild cognitive impairment,” “Wallerian degeneration,” and “neuropathology.” We also hand-searched articles cited in the selected papers, so that publications missed by the electronic research could be added. Inclusion criteria were as follows: original articles written in English and focusing on AD and MCI and DTI studies carried out through TBSS with non-FA indices (see further explanation below) in addition to FA calculation. Reviews and case reports were excluded from this review; studies using clinical constructs other than AD and MCI were also excluded from this study.

### 2.1. Pathological Mechanisms Underlining Retrogenesis and Wallerian Degeneration Model

Evidence based on human neuropathological studies suggests that the brain regions most metabolically active in AD might be also the most capable to respond to mitogenic stimulus and, consequently, those with highest vulnerability to degenerate [16]. One useful terminology for characterizing the pattern of neuronal vulnerability for retrogenic process is the arboreal entropy. According to this model, the greater the neuroprotection, the less vulnerable the myelin and axon. Conversely, neuronal fibers may be attacked from their inside by neurofibrillary and neurotubular changes secondary to hyperphosphorylation, which ultimately may lead to axonal injury and myelin loss [16].

Myelin may be a living, metabolically active part of the neuronal axon, with a membrane running through it, which is an extension of the cell (axonal membrane). Mitogenic activation is involved in cell plasticity and there is consistent evidence showing that mitogenic pathways in neurons are erroneously activated early during AD [26]. Distinct mechanisms may be associated with such mitogenic pathways [26], including hypoxia and *β*-amyloid deposition [27, 28], deficiency of vitamin B12 levels or folate, increased serum homocysteine levels, and increased serum methylmalonic acid levels [29, 30], even though their interaction in the myelin degeneration awaits further elucidation (for a thorough review see Arendt [26]). Atherosclerosis and cerebrovascular disease are other risk factors associated with AD, which have been primarily associated with myelin disruption. Therefore, the entire retrogenesis process implicated in AD neuropathology may comprise myelin, as well as the neuronal reactivation of mitogenic factors. The process of myelination is now known to continue well into the latter portion of life [17, 31]. Possibly, myelin plays a role not only in the conduction of electrical impulses in the neuron, but also in protection and maintenance of the oligodendroglia, myelin, and axonal relationship [17, 31]. Accordingly, early-myelination neurons may become increasingly more thickly myelinated across the years. Consequently, the most recently affected and, as a result, most thinly myelinated brain regions may be the most vulnerable to injury.

**Figure 2:**
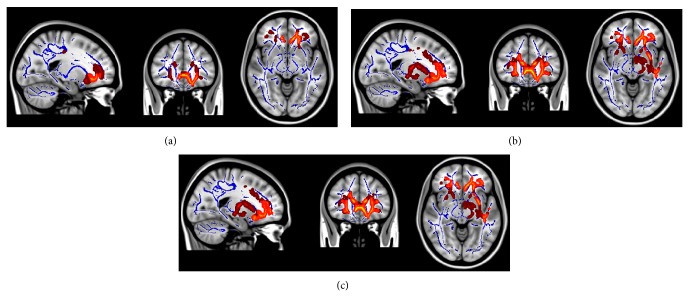
DTI changes are evidenced in Alzheimer subjects when compared with healthy controls. Overlapping areas of FA decreased/DR increased are indicative of increased diffusion perpendicular to fibre orientation, possibly due to myelin breakdown (yellow-red). These areas can be observed in the corpus callosum (anterior and middle segments), anterior cingulum, and uncinate fasciculus (anterior portion) and remain when adjusting for group differences in gray matter atrophy (a) and white matter burden volume (c). Notes: FA: fractional anisotropy; DR: radial diffusivity.

There is consistent evidence suggesting that WM alterations could reflect Wallerian degeneration as a secondary product of cortical pathology [22]. The pathological basis for investigating Wallerian degeneration has been largely demonstrated by experimental animal models, such as those with the sciatic nerve of the frog [32]. In fact, amyloid deposition around neuronal cells or neurofibrillary tangles in the cell bodies ultimately leads to degeneration of axons and myelin [33]. Structural changes including the breakdown and dissolution of both the axonal cytoskeleton and myelin and ultimately the elimination of myelin and other debris by Schwann cells and macrophages are pathological events involved in the secondary degeneration, which is in turn induced by amyloid deposition [32, 34, 35]. Conversely, primary damage to WM tracts has been pointed out by recent studies as an alternative explanation for WM disruption (Figure 2). Interestingly, A*β* deposits around WM vascularity [36] and cellular cytotoxicity provoked by A*β* peptides in oligodendrocytes, the cells responsible for myelin production, have also been reported [37]. Finally, a third mechanism of WM degeneration involving tau has more recently been proposed. The tau protein seems to participate in the integrity and stabilization of axonal cytoskeleton by binding to microtubules [38]. Axonal extensions may become swelled [39] and axonal transport may be disrupted with the functional failure of tau [32, 40]. An important point to be discussed is the relationship of tau production to neuroplasticity. Initial changes in AD may be identified in the entorhinal transitional neuronal networks, which projects through the perforant path to the dental gyrus [19, 41, 42]. Recent studies have demonstrated the mechanism through which tau pathology initially progresses from distal axons to proximal dendrites. Only at later stages may the basal trunk of the dendrites' tree and the body of the neuronal cell be damaged by hyperphosphorylated tau [42]. These events are most likely to be involved in AD pathophysiological cascade.

In summary, the progression of amyloid deposition and hyperphosphorylated tau may hypothetically be linked to the synaptic disconnection of late myelination fibers [42]. Hence, according to the retrogenesis model, small diameter late-myelinating axons of cortical areas would be the earliest and most affected in AD, thereby increasing the susceptibility to amyloid accumulation and hyperphosphorylated tau; conversely, heavily myelinating axons would be less susceptible to AD pathology [17].

### 2.2. Clinical Basis of DTI

As an indirect measure of various aspects of tissue integrity, DTI signal may be influenced by distinct fiber components, including membrane intactness and myelin density [35, 43]. Diffusivity represented by the water motion in a particular region can thus be altered by ordered structures such as axonal tracts in nervous tissues [44]. Diffusivity oriented by the fiber direction, the so-called anisotropic diffusion, is largely restricted in the GM; an increase in anisotropic diffusion may correlate with myelin sheath content, being a valuable parameter for the investigation of WM microstructure integrity [45]. A representation of the ellipsoid can be computed by sampling the diffusivity along multiple directions spaced on a sphere [46, 47]. DTI uses measures derived from the eigenvectors, represented by eigenvalues, which define the diffusion ellipsoid in every voxel [47]. Axial diffusivity (DA) reflects the diffusion coefficient along the principal eigenvector (*λ*_1_), whereas radial diffusivity (DR) indicates the average diffusion coefficients along the two axes perpendicular to *λ*_1_. Mean diffusivity (MD) is a measure of the total amount of diffusion within a voxel and is computed as an average of all three diffusion axes [47]. Finally, FA is a scalar value between zero and one and it is calculated from the eigenvalues (*λ*_1_, *λ*_2_, *λ*_3_) of the diffusion tensor [47] (Figure 1); FA measures the overall directionality of water diffusion and reflects the complexity of cytoskeleton architecture, which restricts the intra- and extracellular water movement [47]. The relationship between FA and WM microstructure changes considerably along the lifespan [32]. Finally, DR measures diffusion perpendicular to the WM fibers while diffusion parallel to the fibers is estimated by DA. MD is considered a nonspecific marker of degeneration which reflects a decrease in membrane or other barriers to free water diffusion.

### 2.3. The Interpretation of DTI Diffusion Indices

The predictive value of conversion to dementia was investigated by a few studies [48]. van Bruggen and colleagues reported higher parameters of Receiver Operator Characteristic curve (ROC) for DR (0.94) and FA (0.94) in both the corpus callosum and left cingulum, while DR and DA in the fornix showed only fair (0.78) indices [48].

Only a few reports investigated regions of overlap between indices [7, 23, 49–52]. Overall, there is still considerable variation among studies in the interpretation of multiple indices [52]. Animal models have proposed that increased MD would be more suggestive of myelin breakdown [53], while an increase in DR or DA is more associated with axonal damage [54]. Most authors describe an increase in DA not accompanied by FA changes as gross tissue loss, widespread tissue damage, and increase of extracellular space [23] which in turn may be a consequence of axonal atrophy secondary to Wallerian degeneration [8, 23]. Conversely, significantly reduced DR without differences in DA has been interpreted as a disruption of myelin integrity in the absence of axonal structural irregularities [8, 15]. These changes would indicate specific damage of the myelin sheaths that restrict DR [8].

Another caveat that restricts the interpretation of diffusion indices is the discrepancy of anatomical findings among DTI investigations. Such constraint may be associated with the different levels of AD severity between participants, which may be responsible for diverse patterns of distribution of DTI changes. While most studies investigated mild to severe individuals [18, 55–57] and mild to moderate participants [7, 50, 58], mild AD patients were investigated by others [4].

### 2.4. Corpus Callosum and Diffusion Alterations

The atrophy of the corpus callosum (CC) has been considered the anatomical correlate of Wallerian degeneration of commissural nerve fibers [4]. The impact of CC atrophy, as predictor of cognitive decline, has been demonstrated in a three-year followup of elders with age-related WM leukoaraiosis [8].

The pattern of neuronal disruption in CC has been discussed by a few studies, but unclear results may rely on the different methods of anatomical parcellation designed for investigation. Based on the Wallerian degeneration hypothesis and on the AD neuronal degeneration pattern [4], earlier stages of WM degeneration should be associated with the involvement of posterior CC subregion, while on later stages the anterior segment of the CC would exhibit atrophic changes [4].

Recent DTI studies have addressed the progression of WM disruption in the CC based on the retrogenesis hypothesis. The CC comprises late-myelinating fibers in the genu [4, 24] and early-myelinating fiber in the splenium. The posterior CC subregion receives axons directly from the temporoparietal lobe, which are the same brain regions primarily affected by AD pathology [24]. Conversely, late-myelinating fibers connect the frontal lobes to the limbic system [52]. One study [59] reported lower FA and higher RD in the body of the CC.

An increasing evidence body has described early DTI changes in the genu of the CC on early (i.e., preclinical) stages of AD. One investigation showed a similar FA profile in the CC between AD and MCI participants who later converted to clinical AD [48]. The CC also exhibited large clusters of voxels with significant differences between MCI converters and nonconverters, especially a decreased FA and an accompanying increase in DR [48]. Taken together, it seems plausible to suppose that in the CC both mechanisms (i.e., myelin breakdown and Wallerian degeneration) may be associated with WM disconnection. Furthermore, these mechanisms may be involved in region-specific illness effects.

## 3. Results

Our review of DTI studies included 11 studies (Table 1). Results are discussed in the following topics.

## 4. Discussion

### 4.1. Regional Differences among DTI Indices

When analysing brain structural and biomolecular changes of AD, some critical points should be taken into account: the characterization of a region specificity, seen as the variability of DTI changes among tracts; the time dependence, defined as the biological processes across different stages of the disease (preclinical and clinical dementia stages); and the hypothetical mechanisms responsible for these modifications in the axonal fibre [8].

According to Brickman and colleagues [60], DTI differences were found in both early- and late-myelinating fibers. In this study, decreased FA and DA and increased DR indices in late-myelinating fibers were proportionally observed, when compared to early-myelinating fibers, in amnestic MCI individuals. These findings provide strong support for the retrogenesis hypothesis. Huang and colleagues also found evidence favouring that WM pathology may be heterogeneous and vary from one tract to another [59]. Hence, the pattern of WM disruption in amnestic MCI takes place initially in limbic and commissural tracts and later on clinically established dementia may progress to the two remaining tracts—projection and association fibers. Similar results for these fibers have been previously reported [11, 59, 61]. These preliminary findings also suggest that cortical atrophy and progression of WM disruption from amnestic MCI to AD may follow a cortical thinning pattern, spreading over time from temporal and limbic cortices to frontal and occipital cortices [59].

The presence of macroscopic WM lesions, often described in the clinical setting as WM burden of vascular origin, may be distinguished from microscopic lesions in terms of brain pathology. Following this assumption, one population-based DTI study [62] reported a few overlapping areas between macro- and micro-WM lesions; instead, distinct areas of macro- and microchanges were found to be predominant [62]. Findings of FA decreases and increases were exhibited in widespread regions, with the fornix being associated with microscopic WM lesions, while periventricular areas were more linked to WM burden [62]. The lack of notable effects of WM burden on DTI findings was also reported by another study [52]. When controlling for the WM burden-effect between AD participants and controls, roughly all areas of anisotropic changes, including MR increases and FA decreases, remained statistically significant [52].

Taken together, the majority of TBSS-based investigations have attempted to establish a pattern of DTI changes that would characterize AD evolution. The sum of evidence regarding a gradient pattern for AD so far remains inconclusive and no firm conclusions can be drawn. On the other hand, whether a gradient of posterior-anterior changes or anterior-posterior changes predominates, or even a combination of these patterns occurring simultaneously, is still under intense debate [7, 8, 15, 23, 49, 52, 58].

### 4.2. Risk Factors for Dementia and Retrogenesis

Cognitive alterations associated with DTI changes might be related with early age alterations during the neurodevelopment stages. For instance, a larger proportion of DTI changes, around 85%, might be related to lower intelligence coefficient (IQ), as pointed by previous investigations [14].

Overall, age has been pointed as one of the most important risk factors for DTI changes [21, 25]. An increasing number of DTI studies indicate that age effects may follow an anterior to posterior gradient on WM changes [49, 60, 63–65]. In one recent investigation, a dissociation pattern of DTI changes was associated with age, with larger effect sizes reported in neocortical late-myelinating fibers in comparison with early-myelinating fibers [60]. These findings are also supported by histological studies, which suggest the development of early-myelinating fibers already at prenatal and perinatal periods, in contrast with the later maturation of neocortical fibers [66, 67]. Moreover, the ageing process may be related to FA alterations, regardless of WM atrophy. Indeed, the decrease of WM volume may fail to show an association with FA changes, as pointed by previous studies [65]. Possibly, only some diffusion properties are prone to affect volume such as the degree of myelination and axon degeneration [25]. In general the cellular microstructure of tissue influences the overall mobility of diffusing molecules and works as intracellular barriers. In summary, the inner properties of FA in the axonal cytoskeleton and microtubules are not fully elucidated and deserve further investigation [35].

Regarding vascular disease, one study showed statistically significant DTI differences between WM hyperintensities (noted by visual scan) and apparent normal WM areas [68]. On the other hand, FA-MD and MD-DR between overlapping areas of macro- and microlesions in WM were interpreted as reflecting demyelination and axonal loss within the fibre and early vascular disease [68].

The presence of a ApoE4 allele was investigated in relation to WM disruption and temporal atrophy in nondemented subjects at risk for AD by Bui and colleagues [69]. FA decreases and DR increases were found in the cingulum, inferior longitudinal fasciculus and inferior frontooccipital fasciculus. Interestingly, no significant MD increases were found. Accordingly, there were no significant correlations between diffusion indices and medial temporal volume. In another investigation, FA decreases and DR increases in the AD group remained after controlling for GM volume [58].

Taken together, these findings strongly suggest that retrogenesis hypothesis may be the driving force behind age-mediated changes for some tracts. However, retrogenesis hypothesis may not fully capture the anatomical changes that occur throughout aging [60]. In fact, well-established late-myelinating fibers, such as the fornix [23] and the superior longitudinal fasciculus [60], may not present overt age-related effects. Finally, taking into account that the interpretation of multiple indices is not clearly established, further studies should comprehensively analyse the application of DTI indices to the understanding of complex interactions between vascular disease and degeneration.

### 4.3. Alternative Hypothesis of Brain Atrophy Progression

In addition to the Wallerian degeneration and retrogenesis hypotheses, a third mechanism of cortical atrophy, in which GM neuronal atrophy may follow axonal damage, has been debated [68]. Previous studies reported higher hippocampal atrophy associated with DTI changes in the fornix and hippocampal tracts (seen as FA decreases combined with DA and DR increases). Conversely, other WM connexions were less associated with hippocampal volume, such as those of periventricular territories [68]. Interestingly, volumetric decreases and DTI changes of WM tract located in the CC (genu and body portions) showed independent effects of age [62]. Additionally, one study involving amnestic MCI participants reported areas less likely to develop overlapping changes due to micro- and macro-WM lesions, among them the fornix and the temporal lobe [12].

The summary of current evidence, although still scarce and preliminary, suggests that particular tracts that are located in temporal and parietal areas may show higher sensitivity to induce cortical atrophy in the surrounding areas and that distinguished and interacting processes, that is, WM atrophy and WM diffusion changes, may be potentially pathologically different.

### 4.4. Limitations of DTI Studies Carried out through TBSS

DTI may be a useful tool for anatomical quantification of microscopic lesions and shed light on the mechanisms of AD pathology, particularly in terms of gradient of progression. Notwithstanding the increasing evidence based on multiple indices studies and the possibility of hypothesising different underlying mechanisms, DTI proxies are not suitable for directly determining the histological background of brain pathology [70, 71]. Hence, multimodal studies incorporating VBM, PET techniques, and conventional neuropathological studies may be necessary to clearly validate DTI parameters. Another awaited achievement is the use of high resolution techniques to assess difficult areas such as the fornix and hippocampus. High resolution DTI is based on optimized sequences for the medial temporal lobe and enables a detailed investigation of each individual fibre bundle to image voxel. In spite of that, DTI alterations in multiple indices may help to elucidate early pathological changes in preclinical stages of AD. The accurate prediction of cognitively healthy individual to convert to clinical AD still remains a significant research challenge. Thus, the idea of a biomarker profile, rather than the single use of one of these techniques, may offer more robust predictive power to determine who is going to convert to AD with acceptable reliability [72]. Another promising use of DTI is the support vector machine approaches, which consists in the statistical analysis of sensitivity and specificity of DTI indices in the differential diagnosis between groups. One study reported a sensitivity of 93% and a specificity of 92.8 in the discrimination between controls and MCI individuals [73] while in other investigations this discrimination yielded a sensitivity of 90.32% and a specificity of 90.41% [74]. One question raised by support vector investigations is the search for the most accurate DTI indices in voxelwise analysis. Statistical significant FA differences between controls and MCI were described by some [23, 51, 52] but not all investigations [7, 8, 75]. Nevertheless, non-FA indices (DA, RD, and MD) failed to show significant results for MCI-control discrimination [8, 51, 52]. Discrepant results may partially be explained on the fact that some studies [7, 23, 52] employed the threshold-free cluster enhancement, which is the most conservative statistical method [76].

Most studies found DA and DR to be more accurate in indicating WM disruption in comparison with FA [23]. However, whether DA or DR increases, but not FA decreases, should be highlighted in the interpretation of DTI findings is still under debate. One has to take into account that DA and DR change in the same direction [51]. As a result, FA changes along fibers may be modified by increases of DA or DR, which may potentially suppress the effect of the changed diffusivity on FA. Such characteristic may also explain major widespread changes in MD and DR, which are absolute diffusion metrics, in comparison to FA [52]. Accordingly, the relation between FA and WM changes presents considerable variations over the disease course, apparently becoming less pronounced on later stages for some WM tracts. A few tracts, like the internal capsule, may exhibit DA increase with no significant change in DR [51].

Another important constraint of DTI studies is the interpretation of multiple indices, based mostly on animal model studies, which lack a consistent pathological validity [23]. For instance, the proper interpretation of DA may be a controversial issue, since both increases and decreases have been reported in the literature [7, 48, 50, 77]. Possibly, the lack of such association might be associated with axonal fibre organization or, alternatively, with DTI calculation in crossing fibre zones, as reported by some studies [23, 78].

Finally, one aspect that remains relevant is the discrepancy between studies concerning techniques of DTI acquisition and processing: the anatomical segmentation for the extraction of DTI values of regional tracts. For instance, the segmentation of CC has generated some controversy regarding the assumed topography of callosal fibers [15]. The other concern is related to partial volume effects, which may underestimate thinner anatomical regions such as the fornix and some limbic structures [48]. Future studies shall incorporate higher resolution MRI and apply automated voxel-based technique (VBM) to overcome these limitations.

Notwithstanding these limitations, multiple diffusion indices approach may be employed as one useful tool for the preclinical diagnosis of dementia. Other biomarkers of risk to dementia, such as the presence of ApoE4 and inflammatory markers (IL6, CRP), may be associated with a steeper decline on cognitive status or greater neuronal loss [79]. Neuroplasticity refers to compensatory and neuroprotective mechanisms which maintain brain structure and activity [80]. The increase in neuronal activity, one of the variables that induce myelination, has been shown to be modulated by plasticity mechanisms which may be extended into old age [79]. Future research on DTI will need to explore how DTI changes would be related to mechanisms of brain atrophy, neuronal compensation, and plasticity.

## 5. Conclusions

The susceptibility of neuronal fibers to the interactions of myelin breakdown, axonal damage, and swelling and other microstructural events may be more deeply appreciated through DTI studies. Moreover, DTI may help mapping the progression of circuit disruption along AD evolution, enabling the establishment of patterns of subclinical features associated with disrupted neuronal pathways. Future neuroimaging studies of dementia will need to transpose with greater accuracy and reliability the complex interpretation of DTI indices, especially on early- and late-myelinating fibers, from animal models to clinical studies. Finally, evidence from DTI provides also a useful surrogate marker of neuronal loss and synaptic disruption and, in addition to cerebrospinal fluid and PET techniques, may be incorporated in the multimodal staging of dementia.

In summary, the use of DTI multiple indices in addition to other standard multimodal methods in dementia research may help to determine the contribution of retrogenesis hypothesis to the understanding of neuropathological hallmarks that lead to AD.

## Figures and Tables

**Table 1:** Main DTI-TBSS studies carried out with multiple indices: AD and control comparisons.

Authors	Sample	Voxelwise contrast	Corpus callosum subregion			Fornix	Temporal lobe	Uncinate fasciculus	Occipital lobe
			Anterior	Middle	Posterior				
Acosta-Cabronero et al., 2010 [50]	AD^1^ (*n* = 25)	AD-controls^∤^	—	↑DA/RD/MD	↑DA/RD/MD	↑DA/RD, ↓FA	↑DA/RD/MD, ↓FA	—	—

Agosta et al., 2011 [76]	AD^3^ (*n* = 23) Controls (n = 15) a-MCI (*n* = 15)	AD-controls^∤^ a-MCI-controls	↑DA/RD/MD ↑DA	↑DA/RD/MD ↑DA	↑DA/MD ↑DA	↑DA/MD, ↓FA —	↑DA/RD/MD, ↓FA ↑DA	↑DA/RD/MD, ↓FA ↑DA	— ↑DA

Alves et al., 2012 [52]	AD^2^ (n = 23) a-MCI (n = 18) Controls (n = 17)	AD-controls^∤^ a-MCI-controls^∤^	↑DA/RD/MD, ↓FA ↓FA	↑DA/RD/MD, ↓FA ↓FA	— —	— —	↑DA/RD/MD, ↓FA ↓FA	↑DA/RD/MD, ↓FA ↓FA	— —

Bosch et al., 2010 [7]	AD^2^ (n = 15) a-MCI (*n* = 16) Controls (*n* = 15)	AD-controls^∤^ a-MCI-controls^∤^	↑DA/DR/MD, ↓FA —	↑DA/MD ↑MD	↑DA/DR/MD —	— ↑MD	↑DR/MD, ↓FA ↑DR	↑DA/DR/MD, ↓FA ↑DR/MD	↑DR/MD, ↓FA ↑DR/MD

di Paola et al., 2010 [8]	AD^1^ (*n* = 38) MCI (*n* = 38) Controls (*n* = 40)	AD-controls^∤^	↑DR, ↓FA	↑DR/DA	↑DR/DA	—	—	—	—

Gold et al., 2010^**^ [70]	High risk ApoE4 (*n* = 37) Low risk ApoE4 (*n* = 20)	High-low risk APOE4^∤^	—	—	—	↓FA	↓FA, ↑DR	↓FA	↓FA

O'Dwyer et al., 2011 [23]	AD^2^ (*n* = 9) a-MCI (*n* = 14) na-MCI (*n* = 19) Controls (*n* = 40)	AD-controls^∤^ MCI-controls^∤^	↑DA/RD/MD, ↓FA ↑MD/DA	↑DA/RD/MD, ↓FA ↓FA	↑DA/RD/MD, ↓FA —	↑DA/RD/MD, ↓FA —	↑DA/RD/MD, ↓FA —	↑DA/RD/MD, ↓FA —	↑DA/RD/MD, ↓FA —

Stricker et al., 2009 [58]	AD^1^ (*n* = 16) Controls (*n* = 14)	AD-controls^λ^	—	—	↓FA	↓FA	↓FA	↓FA	↓FA

Salat et al., 2010 [49]	AD^2^ (*n* = 20) Controls (*n* = 54)	AD-controls NA^∤^	↓FA, ↑DA/DR	—	↑DA/DR	↑DA	↓FA, ↑DA/DR	—	↓FA, ↑DA/DR

Shu et al., 2011 [51]	AD^3^(*n* = 16) a-MCI (*n* = 17) Controls (*n* = 19)	AD-controls^∤^ MCI-controls^∤^ MCI versus AD^∤^	↑MD/DA/DR, ↓FA ↓FA ↑MD	↑MD/DA/DR, ↓FA — —	↑MD/DA/DR, ↓FA ↓FA ↑MD,	↑MD/DA/DR — —	↑MD/DA/DR, ↓FA ↑MD	↑MD/DA/DR, ↓FA ↓FA —	↑MD/DA/DR, ↓FA ↓FA ↑MD

Vernooij et al., 2008 [63]	832 patients from the community	MCI-controls^*^	↓FA, ↑DA/DR	↓FA	↓FA, ↑DA/DR	↓FA, ↑DA/DR	↓FA, ↑DA/DR	—	↓FA, ↑DA/DR

Note: DA: axial diffusivity; MD: mean diffusivity; DR: radial diffusivity; a-MCI: amnestic mild cognitive impairment; na-MCI: nonamnestic mild cognitive impairment; AD: Alzheimer's disease. Method of voxelwise contrast: ^(∤)^familywise error rate; ^(*λ*)^permutation based approach; ^(∗)^not informed. Alzheimer's clinical severity: ^(1)^mild; ^(2)^mild to moderate; ^(3)^severe; ^(∗∗)^only female subjects included.

## Abbreviations

AD: Alzheimer's disease
CC: Corpus callosum
DA: Axial diffusivity
FA: Fractional anisotropy
GM: Gray matter
MD: Mean diffusivity
MCI: Mild cognitive impairment
DR: Radial diffusivity
DTI: Diffusion tensor imaging
WM: White matter
TBSS: Tract-based spatial statistics.
